# Supplementary material for: Lorentz‐Boost‐Driven Magneto‐Optics in a Dirac Nodal‐Line Semimetal
Source: Adv Sci (Weinh). 2022 Jun 17;9(23):2105720. doi: 10.1002/advs.202105720 (PMC9376811; doi:10.1002/advs.202105720)
Supplement: Supplementary file 1 — Supporting Information [file ADVS-9-2105720-s001.pdf]

ADVANCED SCIENCE
Open Access

Supporting Information

for *Adv. Sci.*, DOI 10.1002/advs.202105720

Lorentz-Boost-Driven Magneto-Optics in a Dirac Nodal-Line Semimetal

*Jan Wyzula, Xin Lu, David Santos-Cottin, Dibya Kanti Mukherjee, Ivan Mohelský, Florian Le Mardelé, Jiří Novák, Mario Novak, Raman Sankar, Yuriy Krupko, Benjamin A. Piot, Wei-Li Lee, Ana Akrap, Marek Potemski, Mark O. Goerbig and Milan Orlita**

Supplementary Information for "Lorentz-boost-driven magneto-optics in Dirac matter"

J. Wyzula,[1] X. Lu,[2] D. Santos-Cottin,[3] D. K. Mukherjee,[2,4] I. Mohelský,[1] F. Le Mardelé,[3] J. Novák,[5] M. Novak,[6] R. Sankar,[7] Y. Krupko,[1,8] B. A. Piot,[1] W.-L. Lee,[7] A. Akrap,[3] M. Potemski,[1,9] M. O. Goerbig,[2] and M. Orlita[1,10,*]

[1]*LNCMI-EMFL, CNRS UPR3228, Univ. Grenoble Alpes, Univ. Toulouse, Univ. Toulouse 3, INSA-T, Grenoble and Toulouse, France*

[2]*Laboratoire de Physique des Solides, Université Paris Saclay, CNRS UMR 8502, F-91405 Orsay Cedex, France*

[3]*Department of Physics, University of Fribourg, Chemin du Musée 3, 1700 Fribourg, Switzerland*

[4]*Department of Physics, Indiana University, Bloomington, Indiana 47405, USA*

[5]*Department of Condensed Matter Physics, Masaryk University, Kotlářská 2, 611 37 Brno, Czech Republic*

[6]*Department of Physics, Faculty of Science, University of Zagreb, 10000 Zagreb, Croatia*

[7]*Institute of Physics, Academia Sinica, Nankang, 11529 Taipei, Taiwan*

[8]*Institut d'Electronique et des Systemes, UMR CNRS 5214, Université de Montpellier, 34000, Montpellier, France*

[9]*Institute of Experimental Physics, Faculty of Physics, University of Warsaw, ul. Pasteura 5, 02-093 Warszawa, Poland*

[10]*Charles University, Faculty of Mathematics and Physics, Institute of Physics, Ke Karlovu 5, 121 16 Prague 2, Czech Republic*

CONTENTS

I. Sample growth and x-ray characterization 1

II. Optical response of $NbAs_2$ at $B = 0$ 2

III. Nodal lines in magnetic field 5
A. Landau levels 5
B. Velocity operators 7
C. Selection rules 7

IV. Infrared magneto-reflectivity technique and complementary magneto-optical data 9

V. Magneto-transport experiments 9

References 11

I. SAMPLE GROWTH AND X-RAY CHARACTERIZATION

$NbAs_2$ single crystals explored in this work were grown using a chemical vapor transport method. The as-grown crystals usually have a several facets with different crystalographic orientations with shiny surfaces suitable for infrared reflectivity experiments. In total, four $NbAs_2$ single crystal samples (No. $1 \dots 4$) were used to collect the magneto-optical data presented in this work and characterized using standard x-ray technique. To this end, Bragg-Brentano diffractometer equipped with Cu x-ray tube, channel-cut germanium monochromator and scintillation detector were employed. The x-ray diffraction patterns of each explored sample and facet are presented in Fig. S1. For the purpose of optical experiments on the (001) facet at $B = 0$, the orientation a and b crystallographic axes was determined using pole diagrams. The magneto-reflectivity data with B oriented perpendicular to the (101) and (607) crystallographic planes were collected using the nearby (403) facet on Sample 1 rotated respectively by 5 and 7° in the (a-c) plane.

* milan.orlita@lncmi.cnrs.fr

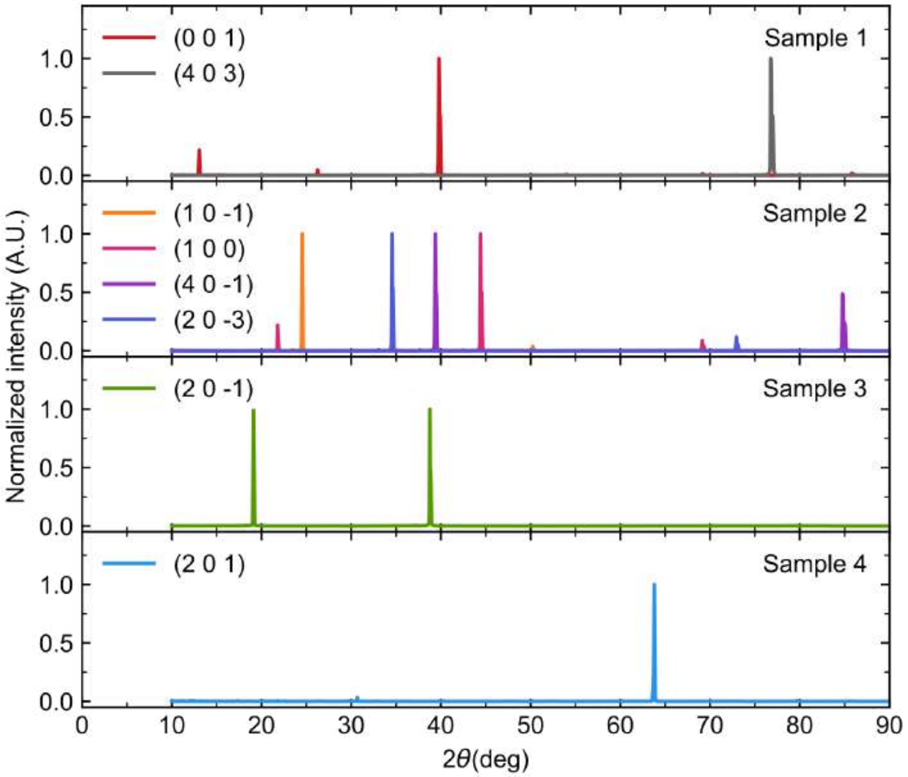

FIG. S1. Normalized diffraction patterns of all facets studied in our magneto-optical experiments. The (001)-oriented facet of Sample 1 was also used for reflectance measurements at $B = 0$.

II. OPTICAL RESPONSE OF $NbAs_2$ AT $B = 0$

To characterize the optical response of $NbAs_2$ at $B = 0$, infrared reflectivity was measured on the (001)-oriented facet using radiation polarized linearly along the a and b crystallographic axes. To this end, the Vertex 70v FTIR spectrometer was used, equipped with custom-built in situ gold evaporation. At high photon energies, the phase was fixed by ellipsometry. Then, the standard Kramers-Kronig analysis was employed to obtain the frequency-dependent complex optical conductivity. The measured reflectivity and the deduced optical conductivity are presented in Fig. 1d in the main part of the manuscript and in Figs. S3a,c,e.

To describe the optical response of $NbAs_2$ at $B = 0$ theoretically, we use the simple model for electronic states in a nodal-line semimetal proposed in the main text. The proposed Hamiltonian, now with both line index $\xi = \pm 1$ and spin included, reads:

$$\hat{H} = (\varepsilon_0 + \hbar w q_{\mathrm{line}})\mathbb{1} + \hbar v(\xi q_x \sigma_x + q_y \sigma_y) + \xi \Delta \sigma_z \tau_z, \tag{S1}$$

where σ_i $(i = x, y, z)$ and τ_z are standard Pauli matrices for orbital and spin degrees of freedom, respectively. This Hamiltonian describes electronic states in the vicinity of any point at the nodal line, $\mathbf{k} = \mathbf{k}_{\mathrm{line}} + \mathbf{q}$. The used orthogonal coordinate system, $\mathbf{q} = (q_x, q_y, q_{\mathrm{line}} \equiv q_z)$, has the third component always aligned with the local direction of the nodal line $\boldsymbol{\tau}$ (see Fig. S2) which is roughly parallel with the a crystallographic axis [1]. The other nodal line is located symmetrically in momentum space with respect to the Γ-Y-Z mirror plane. Similar to the local direction $\boldsymbol{\tau}$, the Hamiltonian parameters ε_0, v, Δ and w also vary, smoothly and weakly along the nodal line, and therefore, can be viewed as a function of q_{line}.

To simplify the problem at hand, we have divided the nodal line zone into four segments I, II, III and IV, which approximately describe its propagation through a half of the Brillouin zone. The locations of these segments, as well as their conduction/valence band energies (at $\mathbf{k}_{\mathrm{line}}$), are shown in Figs. S3b,d,f. The suggested profile of the nodal lines approach the results of DFT simulations and overall conclusions presented by Shao et al.[1]. In each segment, the nodal line is reduced down to a straight line in momentum space – having the length of k^i – and characterized by a set of corresponding parameters: ε_0^i, v^i, Δ^i and w^i, where $i =$ I, II, III and IV, and two angles θ_a^i and θ_b^i which

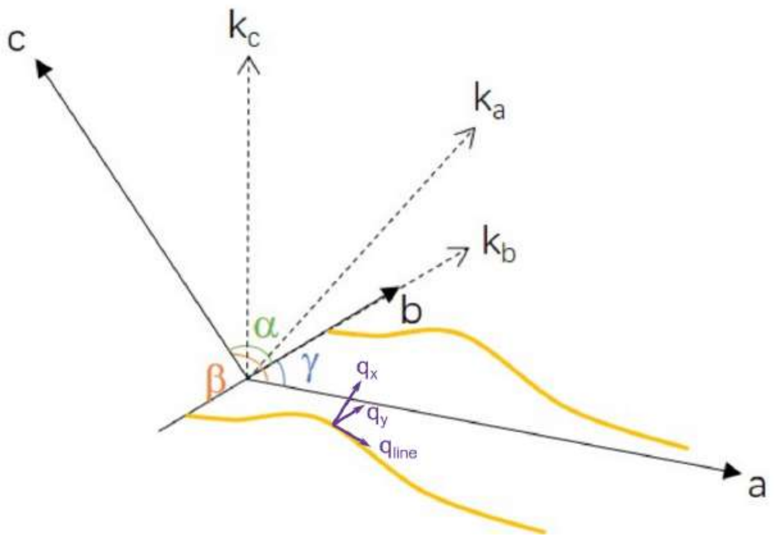

FIG. S2. The position of two nodal lines in $NbAs_2$ with respect to the real space crystal axes a, b, c and the corresponding reciprocal space axes k_a, k_b, k_c. The local axes along the nodal line: $\mathbf{q}_{\rm line}$ is parallel to the nodal line and $\mathbf{q}_{x,y}$ is always orthogonal to it.

encodes the local direction of the given segment $\boldsymbol{\tau}^i$ with respect to a and b crystallographic axes, cf. Figs. S3d,f. Within each segment, these parameters are supposed to be constant except $\Delta^{\rm III}$ which we consider to vary linearly with $q_{\rm line}$ to ensure the continuity of the nodal line in energy.

With such simplifications, the diagonal component of optical conductivity is obtained by integration along the chosen segment i, using the local basis of the Hamiltonian:

$$\Re\left[\sigma^i_{xx}(\omega)\right] = \frac{Ne^2}{8h}\int dq_{\rm line}\left(1+\frac{4(\Delta^i)^2}{(\hbar\omega)^2}\right)[f(\epsilon^i_0+\hbar w^i q_{\rm line}-\hbar\omega/2)-f(\epsilon^i_0+\hbar w^i q_{\rm line}+\hbar\omega/2)]\Theta(\hbar\omega-2\Delta^i) \quad \text{(S2)}$$

and, by the isotropy of the model, $\sigma^i_{xx}(\omega)=\sigma^i_{yy}(\omega)$. The other diagonal component reads:

$$\Re\left[\sigma^i_{zz}(\omega)\right] =$$
$$\frac{Ne^2}{4h(v^i)^2}\int dq_{\rm line}\left(\frac{\partial\Delta^i}{\partial q_{\rm line}}\right)^2\left[1-\left(\frac{\Delta^i}{\hbar\omega}\right)^2\right][f(\epsilon^i_0+\hbar w^i q_{\rm line}-\hbar\omega/2)-f(\epsilon^i_0+\hbar w^i q_{\rm line}+\hbar\omega/2)]\Theta(\hbar\omega-2\Delta^i). \quad \text{(S3)}$$

In the equations above, $N=2$ is the number of the nodal lines in the Brillouin zone (double degeneracy due to spin already included), f is the Fermi-Dirac distribution and Θ is the Heaviside function. The main contribution to the optical conductivity comes from Eq. (S2). Eq. (S3) leads to a non-zero contribution to optical conductivity only when the gap parameter changes with $q_{\rm line}$. Such a possibility is not explicitly included in the Hamiltonian (S1), nevertheless, as mentioned above, it is relevant in our approximation for the segment III (see Fig. S3b). Anyway, in practice, the contribution from (S3) is negligible due to the small ratio between $\hbar^{-1}\partial_{q_{\rm line}}\Delta^i$ and v^i.

In our reflectivity measurements at $B=0$ (Fig. 1d top in the main text), the incident radiation was polarized linearly along the a or b axes. So, to calculate the experimentally probed optical conductivities $\Re\left[\sigma_{aa}(\omega)\right]$ and $\Re\left[\sigma_{bb}(\omega)\right]$, one has to make a corresponding projection for each segment (i =I, II, III and IV) and sum their contributions:

$$\Re\left[\sigma_{aa}(\omega)\right] = \sum_i\left(\cos^2\theta^i_a\Re\left[\sigma^i_{zz}(\omega)\right]+\sin^2\theta^i_a\Re\left[\sigma^i_{xx}(\omega)\right]\right) \quad \text{(S4)}$$

$$\Re\left[\sigma_{bb}(\omega)\right] = \sum_i\left(\cos^2\theta^i_b\Re\left[\sigma^i_{zz}(\omega)\right]+\sin^2\theta^i_b\Re\left[\sigma^i_{xx}(\omega)\right]\right). \quad \text{(S5)}$$

Even though we use a relatively crude approximation for the profile of the nodal lines, there still remains a number of free parameters to be tuned/set. For each segment, we have its length k_i in momentum space, four Hamiltonian parameters v^i, Δ^i, w^i and ε^i_0, two angles θ_a and θ_b. The Fermi energy is an additional parameter common to all segments. To reduce this number, let us consider the following points: (i) When the anisotropy of the dispersion

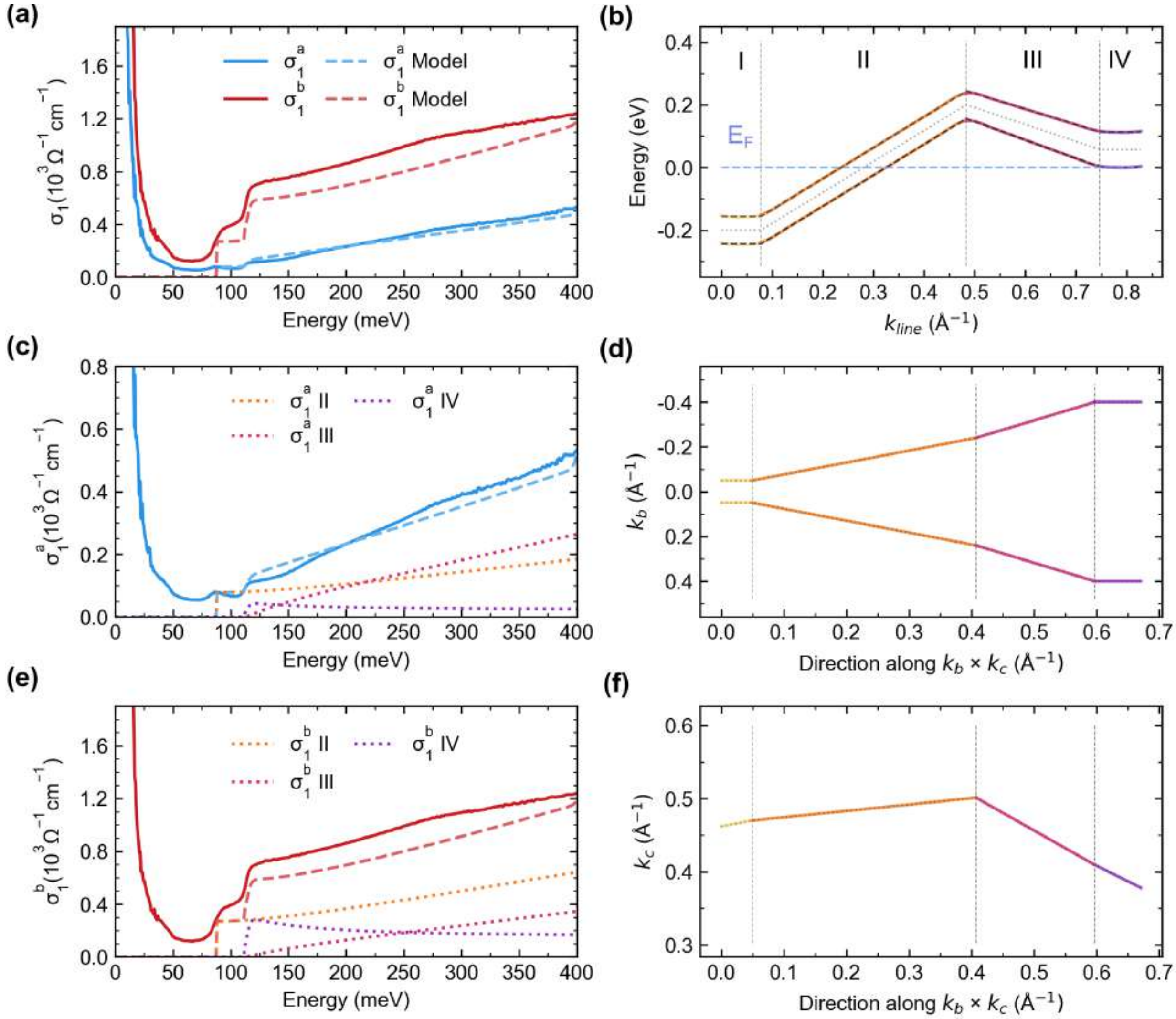

FIG. S3. Parts (a),(c) and (e): Real part of optical conductivity (solid lines) deduced via Kramers-Kronig analysis of the $NbAs_2$ reflectivity response measured on the (001)-oriented facet at $T = 10$ K using radiation polarized linearly along the a and b crystallographic axes. The dashed and dotted lines lines show results of theoretical modelling described in the text. Parts (b), (d) and (f): Energy (at $\mathbf{k}_{\rm line}$) and momentum profiles of the nodal lines considered in the simplified (segment-based) model for optical conductivity of $NbAs_2$ at $B = 0$.

perpendicular to the nodal line is neglected (i.e., $v_x^i = v_y^i$), the main contribution to optical conductivity (S2) becomes independent of the velocity parameter v^i. (ii) For the parameters Δ^i and w^i as well as the corresponding angles, θ_a^i and θ_b^i, for $i =$II and IV, we have solid estimates coming from the analysis of the magneto-optical response, due to the dispersive and flat parts of the nodal line, respectively: e.g., $\Delta^{\rm II} = \Delta_D$, $\Delta^{\rm IV} = \Delta_F$, $w^{\rm II} = w$, $w^{\rm IV} = 0$. (iii) Additional constraints on w^i, Δ^i, ε_0^i and k^i, appear because the nodal lines are continuous in momentum as well as in energy. For instance, the size of the Brillouin zone approximately fixes the sum of segments lengths and the gap parameter $\Delta^{\rm III}$ has to evolve smoothly from $\Delta^{\rm II} = \Delta_F$ to $\Delta^{\rm IV} = \Delta_D$. In the latter case, we choose the simplest linear in $q_{\rm line}$ approximation. (iv) The expected profile of the nodal line [1] allows us to neglect – due to Pauli occupation effect – the contribution of interband excitations coming from the segment I. This is because we are only interested in the optical response at relatively low photon energies (below $\hbar\omega \approx 400$ meV, see Fig. S3a).

Using the constraints (i) to (iv), the number of freely tunable parameters is strongly reduced, basically down to the segments' lengths. The theoretically calculated conductivity approaches fairly well the experimentally measured curves, see Fig. S3a. The contributions of individual segments are shown using dotted lines and the corresponding color coding in Figs. S3c,e. Notably, the agreement could be further improved by considering velocity anisotropy ($v_x^i \neq v_y^i$) which alters the relative strength of individual segments. Such an approach has been successfully used by

Shao et al. [1], nevertheless, we prefer not introducing (three) additional tuning parameters which cannot be directly determined from our magneto-optical experiments.

The obtained values of the used tuning parameters – the lengths of the segments k^i and the angles $\theta_a^{\rm III}$ and $\theta_b^{\rm III}$ – are visualized graphically in Figs. S3d,f in which the nodal line location was projected to the (a-c) and (b-c) crystallographic planes, respectively. Let us remind that the vectors $\mathbf{k_b}$ and $\mathbf{k_b} \times \mathbf{k_c}$ lies in the a-b plane while the vectors $\mathbf{k_c}$, $\mathbf{k_a}$ as well as $\mathbf{k_b} \times \mathbf{k_c}$ lie in the a-c crystallographic plane, see Fig. S2.

III. NODAL LINES IN MAGNETIC FIELD

To model a nodal line subjected to an externally applied magnetic field, we use the (zero-field) Hamiltonian introduced, together with all its parameters, in the main text:

$$\hat{H} = (\varepsilon_0 + \hbar w q_{\rm line})\mathbb{1} + \begin{bmatrix} \Delta & \hbar v(q_x - iq_y) \\ \hbar v(q_x + iq_y) & -\Delta \end{bmatrix}, \tag{S6}$$

where we consider only one spin sector (spin up) of one of the two nodal lines ($\xi = +1$) [cf. Eq. (S1)]. The calculations for spin down or the other nodal line follow exactly the same procedure as we are about to show below.

A. Landau levels

The Hamiltonian (S6) is isotropic in the x-y plane, hence, we may set, with no loss of generality, that $\mathbf{B} = B(\cos\theta \mathbf{e}_{\rm line} + \sin\theta \mathbf{e}_x) = B\mathbf{e}_B$ for $0 < \theta < \pi/2$ and define a new orthogonal basis with the third component aligned with the magnetic field:

$$\{\mathbf{e}_{x'}, \mathbf{e}_{y'}, \mathbf{e}_{z'} \equiv \mathbf{e}_B\} = \{\cos\theta \mathbf{e}_x - \sin\theta \mathbf{e}_{\rm line}, -\mathbf{e}_y, \cos\theta \mathbf{e}_{\rm line} + \sin\theta \mathbf{e}_x\}. \tag{S7}$$

When this new basis is used, the Hamiltonian reads:

$$H = \hbar w\left(q_B \cos\theta - q_{x'} \sin\theta\right) + \hbar v\left(q_{x'} \cos\theta + q_B \sin\theta\right)\sigma_x - \hbar v q_{y'} \sigma_y + \Delta\sigma_z, \tag{S8}$$

and our goal is to find the spectrum in the presence of a magnetic field.

Let us first illustrate the relativistic properties embedded in the above Hamiltonian, which will inspire the solution of the problem. When the dispersion along the direction of the magnetic field is neglected ($q_B = 0$), the Hamiltonian becomes that of a tilted 2D massive Dirac electron

$$H = \hbar w q_{x'} \sin\theta + \hbar v q_{x'} \cos\theta \sigma_x - \hbar v q_{y'} \sigma_y + \Delta\sigma_z, \tag{S9}$$

where the tilt velocity along the x' direction is $w\sin\theta$ and the Fermi velocity along the x' and y' directions are $v\cos\theta$ and $-v$, respectively. However, this Hamiltonian is not that for the Lorentz-invariant Dirac equation but a variant of it due to the tilt term. The relativistic properties are revealed once the magnetic field is introduced using the standard Landau gauge, $\mathbf{A} = -By'\mathbf{e}_{x'}$, so that

$$H - \hbar w q_{x'} \sin\theta = -\hbar w \sin\theta eBy' + \hbar v \cos\theta(q_{x'} - eBy')\sigma_x - \hbar v q_{y'} \sigma_y + \Delta\sigma_z, \tag{S10}$$

where one can identify an effective electric field $Bw\sin\theta$ in the y' direction. Most importantly, the right hand side is now that of the standard Lorentz invariant Dirac equation except the anisotropy of the speed of light in the x' and y' directions. Therefore, one encounters now the problem of a relativistic 2D massive Dirac electron in the presence of an electric field of $Bw\sin\theta$ in the y' direction and a magnetic field of B in the z' direction, thus moving with a drift velocity of $w\sin\theta$ in the x' direction assuming a small drift velocity. Remark that the speed of light in the x' direction is replaced by $v\cos\theta$, so the rapidity is defined as $\beta = w\tan\theta/v$. It is well-known in the theory of special relativity that one can always shut down the electric field in the co-moving frame of an electron subjected to a crossed electric and magnetic field if the drift velocity is smaller than the speed of light, i.e, $\beta < 1$ [2, 3]. Working in the co-moving frame, with the drift velocity, and using a Lorentz boost, the problem is thus simplified to that of an electron subjected only to magnetic field, i.e., the usual Landau quantization problem. Once one finds the Landau levels (LL) in the co-moving frame, the spectrum in the lab frame, i.e., the original frame of reference, follows immediately by doing the inverse Lorentz boost from the co-moving frame back to the lab frame. Technically, this can be conveniently accomplished using hyperbolic transformation, which is the 2D representation of Lorentz boost in the Lorentz group.

In our case, the hyperbolic transformation of the aforementioned Lorentz boost reads $M = \exp(\phi\sigma_x/2)$, with the rapidity $\beta = \tanh\phi = \omega\tan\theta/v$.

Now we are ready to solve the problem described the Hamiltonian below

$$\begin{aligned} H = &\hbar w\left[q_B\cos\theta - (q_{x'} - eBy')\sin\theta\right] + \hbar v\left[(q_{x'} - eBy')\cos\theta + q_B\sin\theta\right]\sigma_x \\ &- \hbar v q_{y'}\sigma_y + \Delta\sigma_z, \end{aligned} \tag{S11}$$

where the magnetic field is already incorporated by the above Landau gauge. Using the same hyperbolic transformation, $M = \exp(\phi\sigma_x/2)$, with the rapidity $\beta = \tanh\phi = w\tan\theta/v$, we obtain the Hamiltonian

$$\begin{aligned} H_T &= MHM \\ &= \hbar\frac{wv}{v^*}q_B + \hbar\frac{w^2+v^2}{v^*}q_B\sin\theta\cos\theta\sigma_x + \hbar v^*(q_{x'} - eBy')\sigma_x - \hbar v q_{y'}\sigma_y + \Delta\sigma_z \end{aligned} \tag{S12}$$

where the velocity v^* is defined as

$$v^{*2} = v^2\cos^2\theta - w^2\sin^2\theta \quad \text{with} \quad \gamma = \frac{1}{\sqrt{1-\beta^2}} = \cosh\phi = \frac{v\cos\theta}{v^*} \tag{S13}$$

where $\gamma > 1$ is the relativistic Lorentz factor.

Note that H_T does not have the same spectrum as H for the reason that we are now working in the co-moving frame. In the basis of $|\psi_T\rangle = \gamma^{-1/2}M^{-1}|\psi\rangle$ given the eigenstate $|\psi\rangle$ of H, one can construct from H_T another Hamiltonian $H_E = (H_T - E\sinh\phi\sigma_x)/\cosh\phi$ with the same spectrum as H

$$H_E = \frac{\hbar w q_B}{\cos\theta} + \frac{1}{\gamma}\begin{bmatrix} \Delta & \hbar\frac{\sqrt{2vv^*}}{\ell_B}a_E^\dagger \\ \hbar\frac{\sqrt{2vv^*}}{\ell_B}a_E & -\Delta \end{bmatrix} \tag{S14}$$

where $\ell_B = \sqrt{\hbar/(eB)}$ is the magnetic length and a pair of energy-dependent ladder operators is defined as:

$$\begin{aligned} a_E &= -\frac{1}{\sqrt{2vv^*}}\left(\frac{v^*}{\ell_B}(y' - \langle y'\rangle_E) + i\ell_B v q_{y'}\right) \\ a_E^\dagger &= -\frac{1}{\sqrt{2vv^*}}\left(\frac{v^*}{\ell_B}(y' - \langle y'\rangle_E) - i\ell_B v q_{y'}\right) \\ \langle y'\rangle_E &= \frac{\ell_B^2}{v^*}\left(v^* q_{x'} + q_B\frac{w^2+v^2}{v^*}\sin\theta\cos\theta - E\frac{w\sin\theta}{\hbar v^*}\right) \end{aligned} \tag{S15}$$

with $[a_E, a_E^\dagger] = 1$. The subscript E indicates the dependence on energy E. In particular, the center of cyclotron $\langle y'\rangle_E$ shifts with energy.

Since the energy-dependent term in H_E is absorbed in the definition of the ladder operators, the energy spectrum is self-consistently found in the eigenstates of energy E_n^λ in the form

$$|\psi_{T,n,\lambda}\rangle = \begin{pmatrix} \cos\zeta_{n,\lambda}|n, E_n^\lambda\rangle \\ \sin\zeta_{n,\lambda}|n-1, E_n^\lambda\rangle \end{pmatrix} \tag{S16}$$

where $\zeta_{n,\lambda}$ is an angle depending on n and the sign of energy $\lambda = \pm$, $|n', E_n^\lambda\rangle$ is the wave function of the one-dimensional quantum harmonic oscillator defined by the previous ladder operators. Given the Landau level index n, $n' = n$ or $n-1$.

Finally, we obtain the LL spectrum:

$$\begin{aligned} E_n^\pm &= \hbar w\, q_B/\cos\theta \pm \sqrt{(\Delta/\gamma)^2 + 2n\hbar eBv^2\cos\theta/\gamma^3} && \text{for } n > 0 \\ E_0 &= \hbar w\, q_B/\cos\theta + \frac{\Delta}{\gamma} && \text{for } n = 0, \end{aligned} \tag{S17}$$

which has the form typical of massive Dirac electrons, nevertheless, with the gap and velocity renormalized by the pseudo-relativistic Lorentz factor: $2\Delta \to 2\Delta/\gamma$ and $v^2 \to v^2/\gamma^3$. Importantly, for the other line index $\xi = -1$ or the spin down we obtain an analogous LL spectrum replacing Δ by $-\Delta$.

It is worth noting that the discussed Lorentz-boost-driven renormalization of the apparent band gap may, to certain extent, resemble the well-known Franz-Keldysh effect [4, 5] which finds its practical use in electro-optical modulation, see *e.g.* Ref. [6]. Even a closer analogy appears when the Franz-Keldysh effect is explored in crossed electric and magnetic fields [7]. Nevertheless, in our case, there is no real electric field applied to the explored system. It only appears in an effective way, in the pseudo-relativistic Dirac-type Hamiltonian, being proportional to the magnetic-field component perpendicular to $\boldsymbol{\tau}_D$.

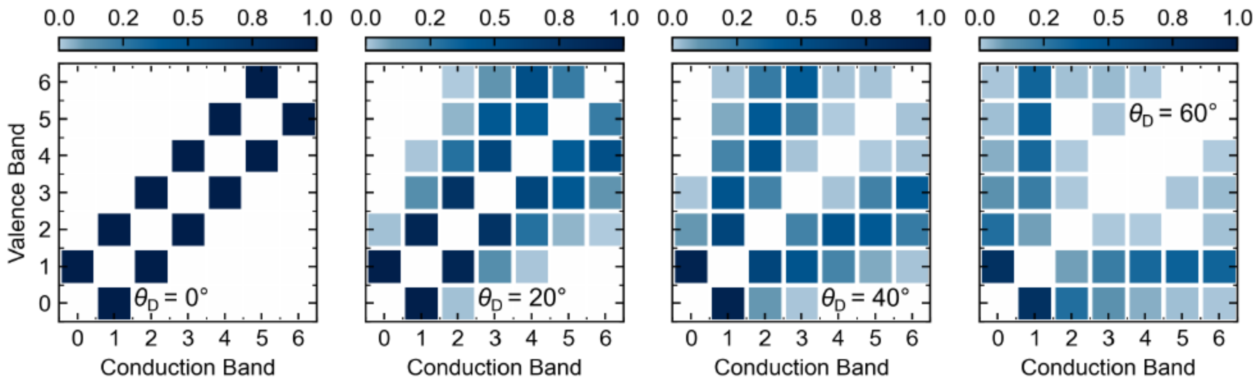

FIG. S4. Relative strength of inter-band transitions between different pairs of Landau levels (for $n = 0 \ldots 6$) plotted for angles $\theta_D = 0$, 20, 40, 60°, and parameters (w, v_D and Δ_D) deduced experimentally for the dispersive part of the nodal line in $NbAs_2$. For $\theta_D = 0°$, we obtain standard selection rules $n \to n \pm 1$ for electric-dipole transitions, typical of all isotropic systems. With increasing θ_D, gradually, additional transitions become allowed ($n \to n \pm 2, \pm 3 \ldots$). For large angles θ_D, but still for $\beta < 1$, one finds a plethora of optically active transitions. The dominant ones follow the rule-of-thumb selection rules $n \to \alpha n$ and $n \to n/\alpha$, where α is an integer ($\alpha = 2 - 4$ for $\theta_D = 60°$).

B. Velocity operators

To study the magneto-optical properties of $NbAs_2$ theoretically, one has to evaluate the matrix element of the corresponding velocity operators: $\langle \psi_n | \hat{v}_{\mathbf{k}} | \psi_m \rangle$. In this case, it is more practical to work with the basis $|\psi_{T,n}\rangle$ already in hands by remarking that

$$\langle \psi_n | \nabla_{\mathbf{k}} H | \psi_{n'} \rangle = \gamma \langle \psi_{T,n} | \nabla_{\mathbf{k}} H_T | \psi_{T,n'} \rangle \tag{S18}$$

thanks to the fact that M is k-independent. So, in the basis $|\psi_T\rangle$, the velocity operators for H_T are

$$\hat{v}_{T,x} = v^* \sigma_x \tag{S19}$$

$$\hat{v}_{T,y} = -v \sigma_y \tag{S20}$$

$$\hat{v}_{T,z} = \frac{wv}{v^*} + \frac{w^2 + v^2}{v^*} \sin\theta \cos\theta \tag{S21}$$

where one notices an emergent anisotropy of the velocity parameter induced by the applied magnetic field.

C. Selection rules

With the velocity operators, one can *a priori* derive the selection rules for electric-dipole inter-LL transitions which are active in the Faraday configuration (the configuration with the wave vector of light parallel to B). We proceed in a way analogous Landau-quantized tilted 3D cones [3]. When calculating the matrix elements such as $\langle \psi_{T,n} | \hat{v}_{T,x} | \psi_{T,m} \rangle$, one has to deal with terms such as $\langle n', E_n | m', E_m \rangle$ which are no longer 0 or 1. This is due to the mismatch of their energy-dependent orbital center [see Eq. (S15)]. Therefore, we do not obtain, in general, the selection rules, $n \to n \pm 1$, typical of isotropic solids [8]. Instead, all direct transitions become in principle possible, as long as the occupation of states (Pauli principle) allows. The transitions stemming from the selection rule other than $n \to n \pm 1$ proliferates in the optical conductivity. By the sum rule, this would dilute the prominent Landau fan which reflects the $n \to n \pm 1$ rule. Therefore, the usual clear-cut Landau fan is blurred.

To illustrate the evolution of electric-dipole selection rules with the angle θ_D between the magnetic field and the local nodal-line direction of the dispersive part $\boldsymbol{\tau}$, we proceed in the way analogous to [3] and [9]. In Fig. S4, we plot normalized squares of matrix elements, such as $|\langle \psi_{T,n} | \hat{v}_{T,x}/v^* | \psi_{T,m} \rangle|^2$, for different interband inter-LL excitations (for indices $n = 0 \ldots 6$) and parameters deduced for the dispersive part of the nodal line in $NbAs_2$. As expected, one obtains the standard selection rules in isotropic systems for $\theta_D = 0°$: $n \to n \pm 1$. This is because the energy-dependence in the cyclotron center is canceled by $\sin\theta$ [see in Eq. (S15)]. For non-zero, but small angles θ_D, the magneto-optical response is still dominated by $n \to n \pm 1$ transitions, although other excitations emerge as well (*e.g.*, $n \to n \pm 2$). In contrast, for larger angles θ_D, one finds a plethora of optical transitions. The dominant ones follow the

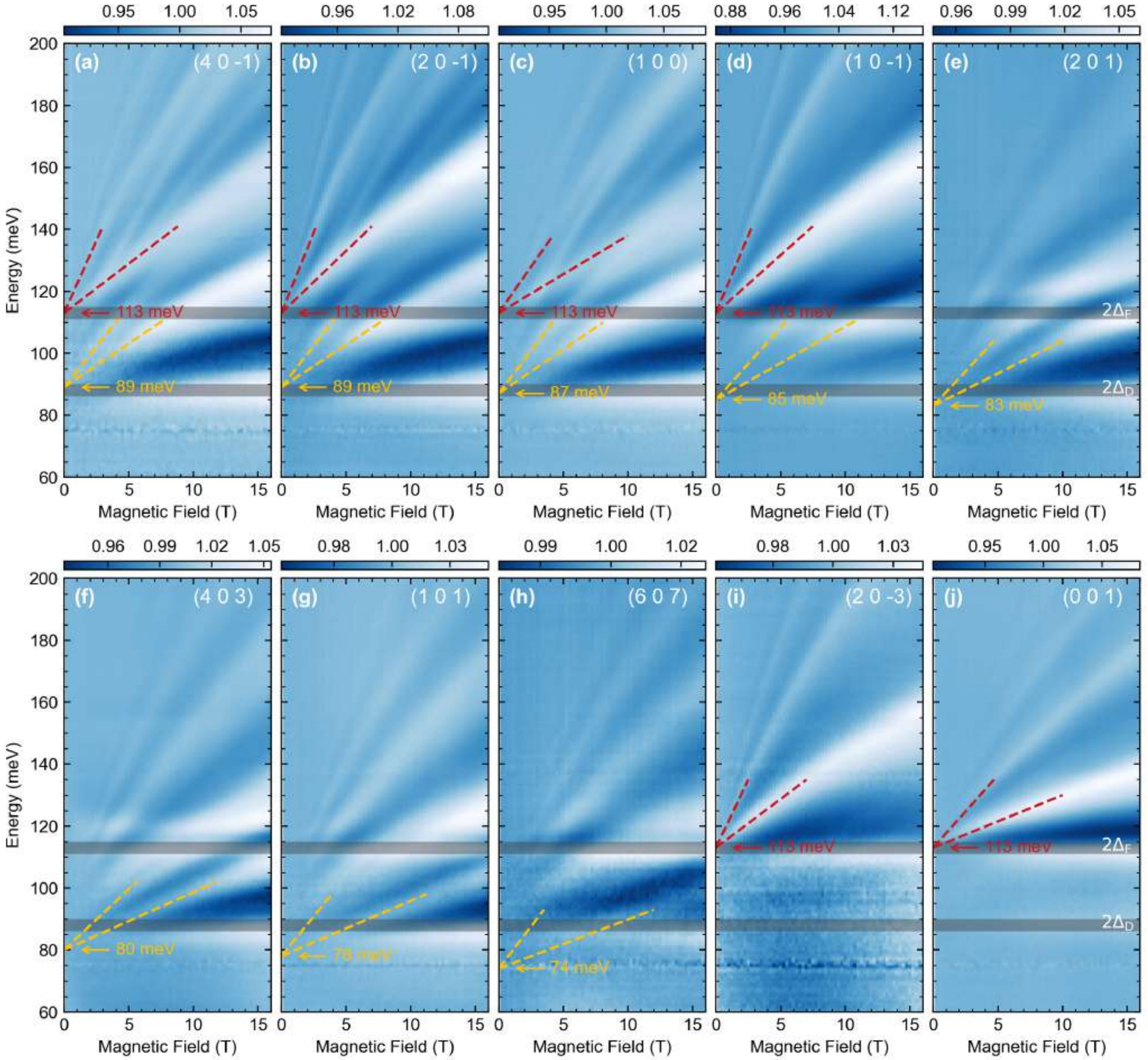

FIG. S5. Parts (a-j): Relative magneto-reflectivity of $NbAs_2$, R_B/R_0, in the magnetic field applied perpendicular to ten different crystallographic planes: $(40\bar{1})$, $(20\bar{1})$, (100), $(10\bar{1})$, (201), (403), (101), (607), $(20\bar{3})$ and (001), which make angles 12, 7, 29, 39, 51, 58, 63, 65, 56 and 90° with the a crystallographic axis, respectively. Horizontal gray bars correspond to positions of two steps in the onset of interband excitations at $2\Delta_D$ and $2\Delta_F$ at $B = 0$, cf. Fig. 1d in the main text or Fig. S3. The yellow and red values indicate the apparent band gap, $2\Delta_D^{\mathrm{eff}}$ and $2\Delta_F$, deduced using a linear zero-field extrapolation of inter-LL resonance belonging to the lower and upper sets (yellow and red dashed lines), respectively.

rule-of-thumb selection rules $n \to \alpha n$ and $n \to n/\alpha$, where α is an integer ($\alpha = 4 - 6$ in the right panel of Fig. S4), in agreement with preceding works on tilted 3D cones [2, 3]. This result may be viewed in a broader context of materials which do not have a full rotational symmetry along the direction of the applied magnetic field and in which inter-LL excitations beyond the basic selection rules $n \to n \pm 1$ become electric-dipole active [10, 11].

IV. INFRARED MAGNETO-REFLECTIVITY TECHNIQUE AND COMPLEMENTARY MAGNETO-OPTICAL DATA

The magneto-reflectivity of $NbAs_2$ was explored in the Faraday configuration, with $\mathbf{B}$ applied perpendicular to the chosen crystallographic plane. During experiments, a macroscopic area of the sample (typically a few mm^2), placed in a superconducting coil and kept at $T = 4.2$ K in the helium exchange gas, was exposed to radiation of a globar, which was analyzed by the Vertex 80v Fourier-transform spectrometer and delivered to the sample via light-pipe optics. The reflected light was detected by a liquid-helium-cooled bolometer placed outside the magnet. The reflectivity R_B recorded at a given magnetic field B was normalized by $R_{B=0}$. To facilitate the data analysis, we assumed that the maxima in relative magneto-reflectivity, R_B/R_0, directly correspond to the positions of inter-LL resonances. This is justified when the imaginary part of the dielectric function exceeds the absolute value of the real part – a condition fulfilled at photon energies around and slightly above the plasma edge (Fig. 1d). A more detailed analysis indicates that, in this way, we slightly overestimate/underestimate the positions of resonances at lower/higher part of the explored range.

In total, we have explored the magneto-optical response of $NbAs_2$ with B applied perpendicular to ten different crystallographic planes. The false-color plots of R_B/R_0 for the (101), (201), (100), $(20\bar{1})$ and (001) planes are presented in the main part of the manuscript (Fig. 2). A complete set of false-colour plots, which include the results collected on all explored facets, is presented in Fig. S5. The corresponding stack-plots of R_B/R_0 spectra are plotted in Fig. S6, except for the $(20\bar{1})$ and (001) facets presented in the main part of our manuscript (Fig. 2). Let us note that the explored $NbAs_2$ monocrystals did not have sufficiently large (101) and (607) facets that would allow us to collect magneto-optical data with a sufficiently high quality. Therefore, we used a larger-in-size (403) facet on Sample 1 rotated respectively by 5° and 7°.

To analyze the magneto-reflectivity data quantitatively, we used the procedure described in the main text. We have focused on the lowest observed line in both sets and assign it to the inter-LL excitation $0 \leftrightarrow 1$ and extracted the effective value of the gap $2\Delta^{\rm eff}$ and velocity $v^{\rm eff}$ using the formula:

$$\hbar\omega_{0\leftrightarrow 1} = \Delta^{\rm eff} + \sqrt{(\Delta^{\rm eff})^2 + 2e\hbar B (v^{\rm eff})^2}. \tag{S22}$$

The extracted values of $2\Delta^{\rm eff}$ and velocity $v^{\rm eff}$ were then plotted for flat and dispersive parts/crossings in Figs. 3a,b and c,d, respectively, in the main part of the manuscript. Notably, for certain facets, inter-LL transitions belonging only to one of two sets are observed. For the upper set, originating in the flat part of the nodal line, this happens for angles θ_F approaching $\pi/2$ and nearly vanishing perpendicular field component $B\cos\theta_F$. This is the case, for instance of the (607) crystallographic plane (Fig. S5h) with $\theta_F \approx 90°$. In contrast, the lower set of inter-LL excitations – stemming from the dispersive part of the nodal line – vanishes at significantly lower angles θ_D, due to Lorentz-boost-driven collapse of the LL spectrum. For instance, the lower set of excitations of completely missing in the response measured with B applied perpendicular to the $(20\bar{3})$ plane, see Fig. S5i, for which $\theta_D \approx 64°$.

V. MAGNETO-TRANSPORT EXPERIMENTS

Magneto-transport experiments provided us with another, complementary characterization of the explored $NbAs_2$ crystals. To this end, we have chosen a monocrystal from the same batch as Sample 1. The electrical contacts were deposited using silver paint in the corners of the (001)-oriented facet of a rectangular shape. The current was always applied along b axis of the sample and the longitudinal magneto-resistance R_{xx} measured at selected (low) temperatures. Two sets of experiments were performed, with the magnetic field applied perpendicular to the a-b plane and along the a axis, see Figs. S7a and b, respectively. In both cases, a pronounced magneto-resistance $R_{xx}(B)$ was observed [12]. Shubnikov-de Haas oscillations became visible in the background removed data ΔR_{xx}. The corresponding fast Fourier-transform applied to $\Delta R_{xx}(1/B)$ implies several characteristic oscillation frequencies and harmonics, see Figs. S7c and d. While the frequency $F_a \approx 300$ T seems to be rather independent of the magnetic field direction, other frequencies, F_b and F_c, indicate a large degree of anisotropy and correspondingly non-spherical Fermi surfaces. The damping of oscillations with temperature has been used to get estimates of the effective (cyclotron) masses m^*. The damping of the most prominent frequencies, i.e., $F_a = 276$ T and $F_c = 120$ T for the magnetic field applied perpendicular to the (001) plane and $F_a = 280$ T with B parallel to the a axis, was analyzed using Lifshitz-Kosevich formula:

$$\Delta_0/\Delta(T_0) = \frac{\alpha T m^*/\bar{B}}{\sinh(\alpha T m^*/\bar{B})}, \tag{S23}$$

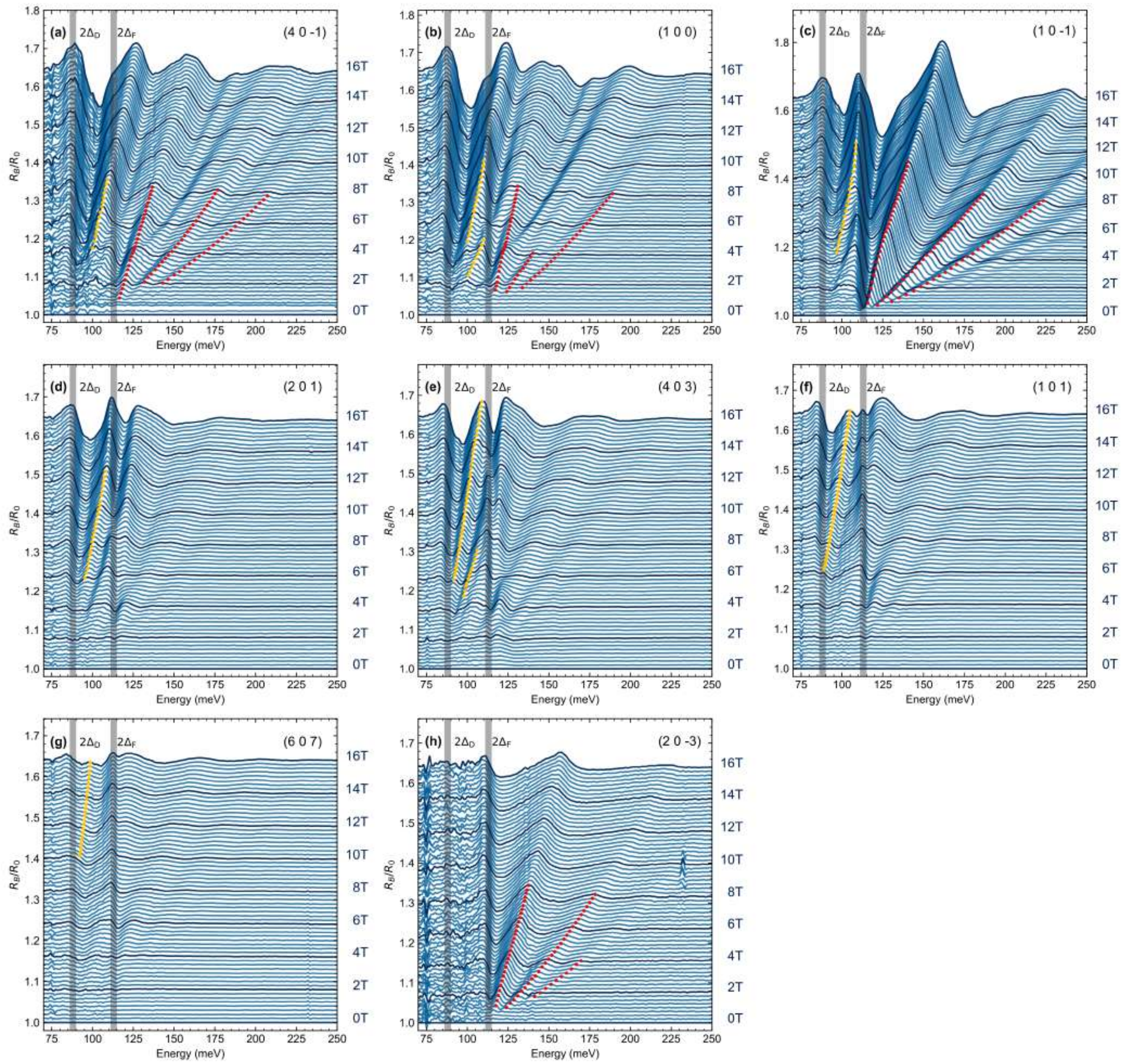

FIG. S6. Parts (a-j): Waterfall plots of relative magneto-reflectivity spectra of $NbAs_2$, R_B/R_0, in the magnetic field applied perpendicular to eight different crystallographic planes: $(40\bar{1})$, (100), $(10\bar{1})$, (201), (403), (101), (607) and $(20\bar{3})$, which make angles 12, 29, 39, 51, 58, 63, 65 and 56° with the a crystallographic axis, respectively. Vertical gray bars correspond to positions of two steps in the onset of interband excitations at $2\Delta_D$ and $2\Delta_F$ at $B = 0$, cf. Fig. 1d in the main text or Fig. S3. The yellow and red points identify maxima of inter-LL resonances belonging to the upper and lower sets at low magnetic fields, respectively.

where $\bar{B}$ stands for the mean magnetic field, T_0 is the lowest measured temperature and $\alpha = 2\pi^2 k_B m_0/(e\hbar) = 14.69$ T/K. The fitted effective masses are similar for all three frequencies $m^* = (0.25 \pm 0.05)m_0$ where m_0 is the bare electron mass. This result is in agreement with values reported in the literature [12–15].

To explore this (an)isotropy in greater detail, we followed the longitudinal magneto-resistance of $NbAs_2$ as a function of the magnetic-field direction with respect to the crystal, while keeping the current flowing along the b axis. In Figs. S7e,f, the magnetic field was applied perpendicular to the a and b axes and the sample rotated around the a and b crystallographic axes, respectively. In the latter case, the frequencies F_b and F_c exhibit a fairly pronounced angle dependence, with the maximum appearing roughly at $\theta_{B,a} \approx 90°$ (i.e., $B \perp a$) and with the maximum-to-minimum

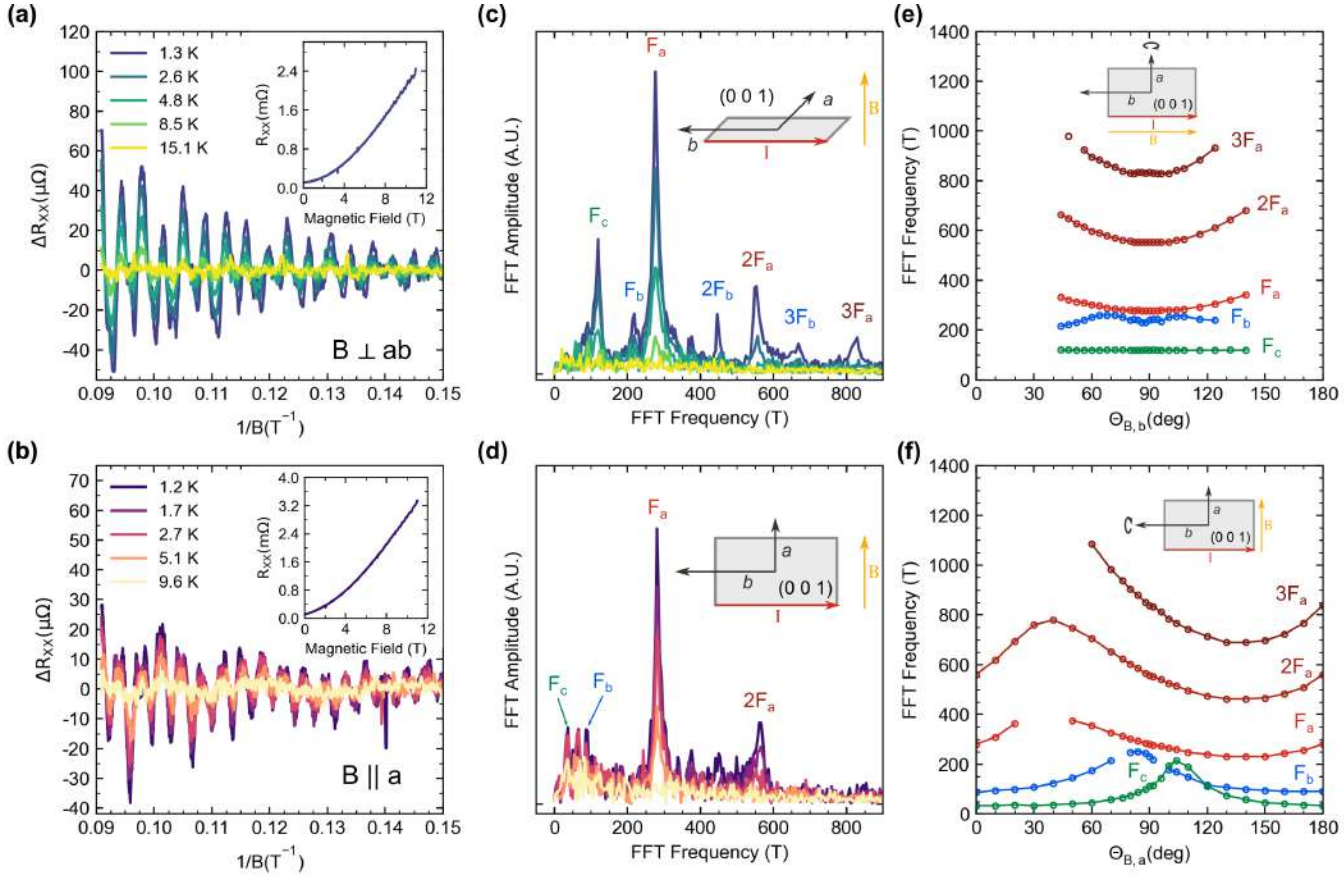

FIG. S7. Parts (a) and (b): Longitudinal magneto-resistance, R_{xx} and $\Delta R_{xx}(B)$, measured at indicated temperatures. The corresponding Fourier-transform applied $\Delta R_{xx}(1/B)$ are plotted in parts (c) and (d): together with insets showing the directions of the magnetic field and current. Parts (e) and (f): Sh-dH oscillation frequencies measured at $T = 1.5$ K as a function of the angle θ between the magnetic field and the b and a axes, respectively, see insets.

ratio reaching nearly 10. This indicates strongly elongated, cigar-like, Fermi surfaces oriented approximately along the a axis. In contrast, the frequencies F_b and F_c remain nearly constant when rotating the sample around the a axis. Such behavior agrees with conclusions of the preceding magneto-transport studies [12–15], and importantly, also with the presence of the nodal lines propagating roughly parallel to the a crystallographic axis.

[1] Y. Shao, Z. Sun, Y. Wang, C. Xu, R. Sankar, A. J. Breindel, C. Cao, M. M. Fogler, A. J. Millis, F. Chou, Z. Li, T. Timusk, M. B. Maple, and D. N. Basov, Optical signatures of Dirac nodal lines in $NbAs_2$, PNAS **116**, 1168 (2019).

[2] S. Tchoumakov, M. Civelli, and M. O. Goerbig, Magnetic-field-induced relativistic properties in type-I and type-II Weyl semimetals, Phys. Rev. Lett. **117**, 086402 (2016).

[3] J. Sári, M. O. Goerbig, and C. Tőke, Magneto-optics of quasirelativistic electrons in graphene with an inplane electric field and in tilted Dirac cones in $\alpha-(\mathrm{BEDT\ TTF})_2\mathrm{I}_3$, Phys. Rev. B **92**, 035306 (2015).

[4] W. Franz, Einfluß eines elektrischen Feldes auf eine optische Absorptionskante, Zeitschrift für Naturforschung A **13**, 484 (1958).

[5] L. V. Keldysh, Behavior of non-metallic crystals in strong electric fields, Soviet Journal of Experimental and Theoretical Physics **6**, 763 (1958).

[6] K. Liu, C. R. Ye, S. Khan, and V. J. Sorger, Review and perspective on ultrafast wavelength-size electro-optic modulators, Laser & Photonics Reviews **9**, 172 (2015).

[7] A. G. Aronov and G. E. Pikus, Light absorption in semiconductors in crossed electric and magnetic fields, Soviet Physics JETP **24**, 339 (1967).

[8] G. Landwehr and E. I. Rashba, *Landau level spectroscopy* (Elsevier, 2012).

[9] The technical trick to analytically evaluate the matrix element is described in the Supplementary information of [2] or

in [16].
[10] M. Orlita, P. Neugebauer, C. Faugeras, A.-L. Barra, M. Potemski, F. M. D. Pellegrino, and D. M. Basko, Cyclotron motion in the vicinity of a Lifshitz transition in graphite, Phys. Rev. Lett. **108**, 017602 (2012).
[11] O. Ly and D. M. Basko, Theory of electron spin resonance in bulk topological insulators Bi_2Se_3, Bi_2Te_3 and Sb_2Te_3, J. Phys.: Condens. Matter **28**, 155801 (2016).
[12] Z. Yuan, H. Lu, Y. Liu, J. Wang, and S. Jia, Large magnetoresistance in compensated semimetals $TaAs_2$ and $NbAs_2$, Phys. Rev. B **93**, 184405 (2016).
[13] B. Shen, X. Deng, G. Kotliar, and N. Ni, Fermi surface topology and negative longitudinal magnetoresistance observed in the semimetal $NbAs_2$, Phys. Rev. B **93**, 195119 (2016).
[14] Y.-Y. Wang, Q.-H. Yu, P.-J. Guo, K. Liu, and T.-L. Xia, Resistivity plateau and extremely large magnetoresistance in $NbAs_2$ and $TaAs_2$, Phys. Rev. B **94**, 041103 (2016).
[15] G. Peramaiyan, R. Sankar, I. P. Muthuselvam, and W.-L. Lee, Anisotropic magnetotransport and extremely large magnetoresistance in $NbAs_2$ single crystals, Sci. Rep. **8**, 1 (2018).
[16] M. O. Scully, M. S. Zubairy, *et al.*, Quantum optics cambridge university press, Cambridge, CB2 2RU, UK (1997).
